# Skin Dosimetry in Radiotherapy of Breast Cancer: a Comparison between EBT and EBT3 Radiochromic Films

**Published:** 2016-06-01

**Authors:** M.T. Bahreyni Toosi, N. Mohamadian, M. Ghorbani, F. Khorshidi, F. Akbari, C. Knaup

**Affiliations:** 1 Medical Physics Research Center, Mashhad University of Medical Sciences, Mashhad, Iran; 2 Medical Physics Department, Reza Radiation Oncology Center, Mashhad, Iran; 3 Comprehensive Cancer Centers of Nevada, Las Vegas, NV, USA

**Keywords:** Film dosimetry, EBT radiochromic film, EBT3 radiochromic film, Breast cancer, radiotherapy

## Abstract

**Objective:**

Radiochromic EBT3 film is a later generation of radiochromic films. The aim of this study is to compare EBT and EBT3 radiochromic films in radiotherapy fields of breast cancer.

**Methods:**

A RANDO phantom was irradiated by a 6 MV Siemens Primus linac with medial and lateral fields of radiotherapy of breast cancer. Dosimetry was performed in various points in the fields using EBT and EBT3 films. Films were scanned by a Microtek color scanner. Dose values from two films in corresponding points were compared.

**Results:**

In the investigation of calibration, net optical density (NOD) of EBT radiochromic is more than the EBT3 radiochromic film. The highest percentage difference between NODs of two films is related to 0.75 Gy and equals to 14.19%. The lowest value is related to 0.2 Gy dose and is equal to 3.31%. The highest percentage difference between two films on the RANDO phantom in breast cancer fields is 13.51% and the minimum value is equal to 0.33%.

**Conclusion:**

From the comparison between the two films, most of the points show differences in dose in the measurements in fields of breast cancer radiotherapy. These differences are attributed to the thickness of the active layers, the overall thickness of the films, and the difference in the calibration fitted functions. The advantage of EBT film over EBT3 is a higher sensitivity; on the other hand EBT3 film allows to use its both sides in the scanning process and it is a new version of this film type.

## Introduction

One of the widely used dosimetry tools is radiochromic film. Characteristics of this dosimeter are sub-millimeter spatial resolution, and low spectral sensitivity (in the range of 0.1-10 Gy)[1] which is desirable in measurement of dose distributions in areas within a radiation field with high dose gradient[2]. These films have effective atomic numbers close to water (7.3)[1] and are used to measure photons and electrons. Other advantages of radiochromic film include, independence from dose rate in range of 0.08-80 Gy/min[3], insensitivity to visible light, easy maintenance and preparation in room light. Development of radiochromic dosimeters is measured directly without any need for chemical processing which shows the amount of radiation. Image formation occurs as color change due to a polymerization process.

Ionization chambers and semiconductors do not provide suitable spatial resolution required for treatment designs in radiotherapy. Thermoluminescent dosimeters, even with small dimensions, are laborious and time consuming for one or two dimensional (2D) dose distribution measurement. Evaluation of ionizing radiation using a radiographic silver halide film is difficult, because it shows high dependence in sensitivity to photon energy in the range of 10 to 200 keV. Energy absorption characteristics of radiographic films do not correspond with soft tissues of the body. Sensitivity to light and the need for controlled chemical processing are other disadvantages of radiographic films. Due to these difficulties, it is necessary to find a dosimeter with high spatial resolution which is associated with fewer difficulties and the ability to provide the amount of absorbed dose with acceptable accuracy and precision as well as associated with the maintenance and data analysis[4].

Dosimetry by radiochromic film is a simple and rapid method for determination of isodose curves and 2D dose distribution as well as in relative dosimetry[5]. Gafchromic EBT films were released in 2004 by International Specialty Products (ISP) company[6]. These films have been accepted as a 2D reference detector by the scientific community[7]. In 2009 EBT2 was released, and in 2011, the EBT3 type was produced. The structure of EBT3 film is symmetrical, like EBT, but in comparison to EBT, it has layers of different compositions with only one sensitive layer[6], while EBT has two sensitive layers[2]. The composition and thickness of the sensitive layer of EBT3 is the same as EBT2 without any difference or priority, but unlike the EBT2, it has symmetrical structure that allows the user to use both sides of the film[6].

So far, various studies were performed in the field of comparison of EBT and EBT3 radiochromic films and also EBT2 and EBT3 radiochromic films. In a comparison between EBT and EBT2 radiochromic films, Andres, et al.[7] have evaluated the sensitivity to light, film response in different color channels, temperature dependence and dependence on the direction of scan. Reinhardt, et al.[1] have performed a study on EBT2 and EBT3 radiochromic films and investigated the dependence to the direction of scan and also film response to photon and electron beams. Few studies have compared the differences between EBT and EBT3 radiochromic films. Furthermore, previous studies have not compared these types of film in real conditions encountered in radiotherapy. For example, in the previous studies measurements were performed on a geometric phantom or on a slab phantom. The aim of this study is to compare EBT and EBT3 radiochromic films in radiotherapy fields of breast cancer, on a RANDO phantom to evaluate and compare the advantages of these film types in a real irradiation situation in radiotherapy.

## Material and Methods

In this study, using EBT and EBT3 radiochromic films, calibration, surface and depth dose measurement in a slab phantom and skin dose on a RANDO phantom were measured. The RANDO phantom was irradiated with medial and lateral tangential fields used in breast cancer radiotherapy by a 6 MV Siemens Primus linac.

### Characteristics of EBT and EBT3 radiochromic films

**Figure 1:**
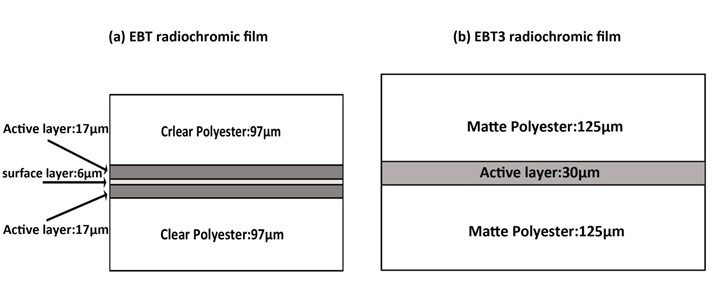
A schematic view of the structures of EBT (a) and EBT3 (b) radiochromic films.

EBT and EBT3 films are two types of radiochromic films that were introduced by the ISP company for dosimetry purposes. Features of these two films, including their structure and color are listed in Table 1. Schematic geometries of these films are illustrated in Figure 1.

**Table 1:** Characteristics of EBT and EBT3 radiochromic films including color and structural features.

**Film type**	EBT	EBT3
**Color of film**	Blue	Yellow
**Structural layers**	Clear polyester (97 µm)	Matte polyester (125 µm)
	Active layer (17 µm)	Active layer (30 µm)
	Surface layer (6 µm)	Matte polyester (125 µm)
	Active Layer (17 µm)	
	Clear polyester (97 µm)	
**overall thickness**	234	280

### Calibration of EBT and EBT3 radiochromic films

In order to calibrate these films, one sheet of EBT radiochromic film from a box (lot number 34351-05) was used which cut into 20 pieces of 2 cm × 3 cm films. These pieces were divided into 10 groups each including 2 pieces. 24 hours before irradiation of these film pieces, they were scanned using a Microtek scanner (ScanMaker 1000XL Pro: Microtek International Inc., Hsinchu, Taiwan) in order to record their background optical density. The scanner was turned on at least 30 minutes before scanning to reduce the error due to heating of the scanner bed, and the readings were performed in 1 minute intervals. In all of the scans, the films were positioned on the center of the bottom-third region of the scanner in portrait mode. Readings were performed at room temperature. In order to record the correct optical density, films must be as much as possible free from scratches and dust. For this purpose, the films were cleaned with a piece of cloth before each scanning.

Films were scanned without any software correction as 48 bit red, green, blue (RGB) color images, as positive, with 100 dots per inch (dpi) resolution. Images were saved in Tagged Image File Format (TIFF) fields. To reduce the effect of scanner noise, each film was scanned 3 times in transmission mode. Since the response in the red color channel is higher than the two other channels, after scanning, the pixel data in red channel were extracted by MATLAB software (version 7.11.0.584, MathWorks, Inc., Natwick, MA). Each group of films was calibrated by Siemens Primus linear accelerator in Reza Radiotherapy and Oncology Center in 10 cm × 10 cm field with different doses (0.2, 0.5, 0.75, 1, 1.25, 1.5, 1.75, 2, 2.5, 3 and 4 Gy) at depth of 10 cm in a PTW solid water phantom with source to surface distance (SSD) of 100 cm. It should be noted that the output of the linac has been calibrated before for therapeutic applications by an ionization chamber. 36 hours after irradiation, the films were scanned similar to the condition in background reading and then, the net optical density (NOD) was extracted by programs written in MATLAB software environment from the following formula:

*NOD*=* OD_Cal_*-* OD_BG_*=-(*log*_10_(* P_Cal_*)-*log*_10_(* P_BG_*))     (1)

where, *OD_Cal_* is calibration optical density, *OD_BG_*is background optical density, *P_Cal_* is the calibration pixel value, and *P_BG_* is background pixel value. Then NOD was plotted versus the given dose and a function was fitted to the corresponding data. All of these steps were repeated in the same condition for EBT3 film (lot number A04011301).

To investigate the light attenuation by unexposed films, five 2 cm × 3 cm pieces of EBT film from the same box used in the calibration were prepared. Initially the scanner bed without any film was scanned, and then each film was scanned separately. The reading of each film was repeated three times. NOD for each film was calculated from the following formula:

*NOD*=* OD_F_*-* OD_NF_*=-(*log*_10_(* P_F_*)-*log*_10_(* P_NF_*))          (2)

where, *OD_F_* is the optical density of film, *OD_NF_* is optical density of the scanner’s bed (no film), *P_F_* is the amount of pixel value of the scanned film and *P_NF_* is the amount of the pixel value of the bed of the scanner. Then, the average of the NOD of the 5 films was calculated. This investigation was repeated in a similar method with EBT3 film.

In order to calculate the total uncertainty of the reported NOD data, the standard deviation for the repeated measurements (including repeated measurements by two films and repeated scanning) was calculated. The standard deviation was considered as type A uncertainty of the measurements. Type B uncertainty was then neglected and the combined uncertainty was assumed to be equal to the type A. Then by multiplication of the cover factor and the combined uncertainty, the expanded uncertainty was obtained and reported. A cover factor of 2.0 was considered, which corresponds to 95.0% confidence interval.

### Surface dose and depth dose measurement in a slab phantom 

In order to measure the surface dose and depth dose in the specified depths in the solid phantom, the EBT and EBT3 radiochromic films and Semiflex ionization chamber (IC) were used. For this purpose, EBT film was cut into 2 cm × 3 cm pieces. Then, each film was put on central axis of a 10 cm × 10 cm field at SSD of 100 cm in a solid water phantom (PTW, acrylic and RW3 slab phantoms) in depths of 0.1, 0.5, 1.5, 2, 3, 4 and 10 cm in such a way that 100 cGy was delivered on the depth of 10.0 cm of the phantom by Siemens Primus linac in Reza Radiotherapy and Oncology Center (Mashhad, Iran). This process was repeated three times in order to increase the reproducibility of the irradiations. Similar to the calibration step, these pieces were scanned by a 1000XL Microtek scanner and then the relevant doses were extracted using the fitted calibration function. This procedure was also performed similarly for the EBT3 films and the results were compared with those of the EBT films. Then, the measured doses were compared with corresponding doses that were obtained from Semiflex ionization chamber. Ionization chamber data had been obtained in a water phantom as part of linac commissioning. The method for calculation of the total uncertainty for the slab phantom measurement data was the same as aforementioned for the calibration step, with the difference that in this step the measurements were repeated for three film pieces for each measurement case and the standard deviation was calculated for the three film measurements.

### Skin dose measurement on RANDO phantom

**Figure 2:**
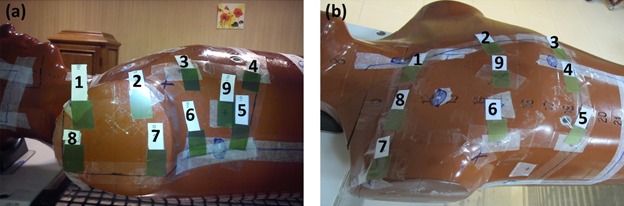
Positions of radiochromic films in (a): the medial field; (b): the lateral field. Each position is remarked by a specific number.

In order to measure the skin dose in different parts of medial and lateral radiation fields in breast cancer radiotherapy, a treatment plan was made by an oncologist. This plan was based on a mastectomy case on a male RANDO phantom. RANDO phantom was used in this study, because it provides a relatively real geometric condition close to the human body for dose measurement in radiotherapy fields. Initially, contouring and determining the borders of the fields was performed for the RANDO (Radiology Support Devices Inc., Long Beach, California, USA) phantom. RANDO phantom was scanned by a computed tomography scanner (Siemens Somatom Emotion Duo) at Reza Radiotherapy and Oncology Center in a horizontal position in such a way that the radiation beam was perpendicular to the body of the phantom. The phantom was scanned totally from its pelvis up to the head. The slice thickness in CT scanning was 0.5 cm.  Using the images from the CT, a breast cancer treatment (mastectomy) was planned using Prowess Panther (version 5.1, Siemens, Germany) treatment planning system. The characteristics of this planned treatment are presented in Table 2. According to this planning, RANDO phantom was then irradiated by Siemens Primus medical linear accelerator. The fields were set up on the phantom, and the 2 cm × 3 cm pieces of EBT and EBT3 films, each from the same box used in calibration were put on the center, corners and middles of the sides of the medial and lateral to cover different positions. In this configuration, the films were fixed in one direction on the phantom by adhesive tape. The configuration of films in the medial and lateral fields is shown in Figure 2. After irradiation of the films, reading procedure was performed the same as explained in the calibration step. The method for calculation of the total uncertainty for the on-RANDO phantom measurement data was the same as described above for the calibration step. However, there was a difference that in this step the measurements were repeated for three film pieces for each measurement case and the standard deviation was calculated for the three film measurements.

**Table 2:** Specifications of the medial and lateral fields in breast cancer radiotherapy used for irradiation of the EBT and EBT3 films.

	**Lateral field**	**Medial field**
Gantry angle (degrees)	55.00	229.00
Couch angle (degrees)	7.00	353.00
Couch (Lat., Vert., Long.) (cm)	5.46, -22.63, 52.79	14.52, -15.63, 52.79
Isocenter (*X*, *Y*, *Z*) (cm)	-5.46, -30.54, 0.16	-14.52, -30.54, -6.84
SSD (cm)	100.00	100.00
Collimator angle (degrees)	356.00	355.00
Field size (cm)	10.00 × 20.00	10.00 × 20.00
Dose at d_max _(Gy)	1.207	1.183

## Results

### Calibration the radiochromic films

The irradiated dose (Gy) and percentage difference between NODs from EBT and EBT3 radiochromic films are listed in Table 3. These data are from the calibration step which were rounded to 4 decimal places. The percentage differences (%) between the NOD values of EBT and EBT3 films were calculated from the following formula:

(*Percentage difference in NOD*)*_EBT_*_ -*EBT*3 _=

100×((* NOD_EBT_*- *NOD_EBT_*_3_)/* NOD_EBT_*)                 (3)

**Table 3:** Obtained NOD values by EBT and EBT3 radiochromic films and the given dose (Gy) in the calibration step and the corresponding percentage differences (%).

**NOD**			
**Radiation dose (Gy)**	**EBT film**	**EBT3 film**	** (Diff.(%))_EBT- EBT3_**
0.20	0.0241±0.0010	0.0233±0.0001	3.31%
0.50	0.0592±0.0000	0.0547±0.0379	7.60%
0.75	0.0930±0.0000	0.0798±0.0001	14.19%
1.00	0.1127±0.0001	0.1019±0.0006	9.58%
1.25	0.1355±0.0000	0.1232±0.0002	9.08%
1.50	0.1540±0.0001	0.1418±0.0002	7.92%
1.75	0.1702±0.0001	0.1582±0.0001	7.05%
2.00	0.1862±0.0000	0.1728±0.0026	7.20%
3.00	0.2430±0.0002	0.2280±0.0003	6.17%
4.00	0.2945±0.0001	0.2705±0.0001	8.15%

**Figure 3:**
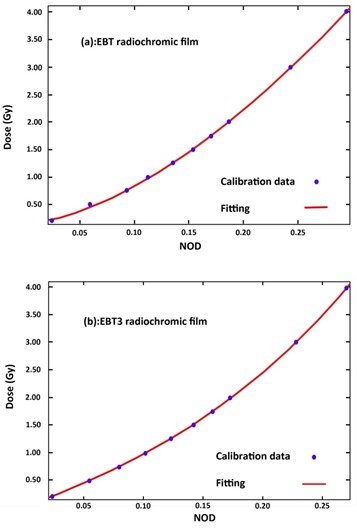
Calibration curve (dose in Gy versus NOD) for radiochromic (a): EBT and (b): EBT3 films.

Figure 3 (a) shows the calibration curve (dose in Gy versus NOD) for Gafchromic EBT film.

The data in this curve was processed by MATLAB software and the following equation was fitted to the data of the absorbed dose (in Gy) as a function of NOD:

*D*=27.38×*NOD*^1.606^+0.1592                        (4)

In this fitting, *R*^2^ =0.9996.

Figure 3 (b) shows the calibration curve (dose in Gy versus the NOD) for Gafchromic EBT3.

The data in this curve is described by the following equation in terms of absorbed dose (in Gy) versus NOD:

*D*=1.152×*e*^ 5.225 *NOD*^-1.138×e^ -1.623 *NOD*^             (5)

In this fitting, *R*^2^ =0.9996.

In the investigation of light attenuation by the films, the difference between the optical density of the EBT radiochromic film and the scanner’s bed is equal to 0.08 and for the EBT3 radiochromic film is equal to 0.04.

### Dose evaluation in slab phantom with EBT and EBT3 radiochromic films

The dose in Gy for various depths in the PTW slab phantom for EBT and EBT3 radiochromic films as well as for Semiflex ionization chamber (IC) with the percentage differences in this measurement are mentioned in Table 4.

**Table 4:** Dose values (Gy) by EBT and EBT3 radiochromic films and Semiflex ionization chamber in PTW slab phantom and the corresponding percentage differences (%).

**Depth (cm)**	**Dose (Gy)**					
	**EBT film**	**EBT3 film**	**Ionization chamber**	** (Diff. (%))_EBT-EBT3_**	** (Diff. (%))_EBT-IC_**	** (Diff. (%))_EBT3-IC_**
0.0	0.27±0.00	0.27±0.00	0.61±0.01	-2.51%	-56.30%	-55.20%
0.5	1.21±0.00	1.21±0.00	1.27±0.01	-0.07%	-4.43%	-4.35%
1.5	1.43±0.00	1.39±0.00	1.46±0.01	2.60%	-2.34%	-4.89%
2.0	1.38±0.00	1.40±0.00	1.45±0.01	-1.65%	-4.77%	-3.20%
3.0	1.33±0.00	1.32±0.00	1.39±0.01	0.68%	-4.09%	-4.74%
10.0	0.91±0.00	0.96±0.00	1.00±0.01	-5.41%	-9.13%	-4.21%

Percentage differences (Diff. (%)) were calculated from the following formula:

(*Diff*.)*_EBT_*_ -*EBT*3 _=100×((* D_EBT_*-* D_EBT_*_3_)/* D_EBT_*)     (6)

(*Diff*.)*_EBT_*_ -*IC*_=100×((* D_EBT_*-* D_IC_*)/* D_IC_*)           (7)

(*Diff*.)*_EBT_*_ 3-*IC*_=100×((* D_EBT_*_3_-* D_IC_*)/* D_IC_*)         (8)

### Skin dose measurement on RANDO phantom

Table 5 lists the skin dose (Gy) in the medial and lateral fields measured in breast cancer treatment using RANDO phantom using EBT and EBT3 radiochromic films. These data are related to the positions which are shown in Figure 2. The percentage differences were calculated based on EBT and EBT3 radiochromic films values which are listed in Table 5.

**Table 5:** Dose results (Gy) in the designated points in the medial and lateral fields of breast cancer radiotherapy using EBT and EBT3 radiochromic films based on the positions in Figure 2.

	**Type of film**	**NOD in medial field**	**NOD in lateral field**	**Dose in medial field (Gy)**	**Dose in lateral field (Gy)**	** (Diff.)_EBT-EBT3_ in medial field **	** (Diff.)_EBT-EBT3_ in lateral field **
Position 1	EBT	0.0886±0.0002	0.0826±0.0003	0.72±0.00	0.66±0.00	-13.51%	-10.62%
	EBT3	0.0859±0.0004	0.0779±0.0000	0.82±0.00	0.73±0.00		
Position 2	EBT	0.0811±0.0000	0.0800±0.0000	0.64±0.00	0.63±0.00	-4.79%	-5.85%
	EBT3	0.0728±0.0002	0.0724±0.0004	0.67±0.00	0.67±0.00		
Position 3	EBT	0.0846±0.0001	0.1211±0.0002	0.68±0.00	1.08±0.00	1.22%	3.83%
	EBT3	0.0724±0.0008	0.1055±0.0007	0.67±0.01	1.04±0.01		
Position 4	EBT	0.0983±0.0002	0.1172±0.0001	0.82±0.00	1.03±0.00	0.85%	-0.33%
	EBT3	0.0857±0.0004	0.1053±0.0004	0.81±0.00	1.04±0.00		
Position 5	EBT	0.1192±0.0000	0.0857±0.0000	1.06±0.00	0.69±0.00	-3.68%	5.49%
	EBT3	0.1103±0.0004	0.0705±0.0001	1.10±0.01	0.65±0.00		
Position 6	EBT	0.1063±0.0008	0.0894±0.0001	0.91±0.01	0.73±0.00	-5.65%	-2.23%
	EBT3	0.0986±0.0003	0.0792±0.0003	0.96±0.00	0.74±0.00		
Position 7	EBT	0.0697±0.0000	0.0771±0.0001	0.54±0.00	0.61±0.00	-4.56%	-2.23%
	EBT3	0.0620±0.0001	0.0669±0.0001	0.56±0.00	0.61±0.00		
Position 8	EBT	0.0966±0.0000	0.0767±0.0001	0.80±0.00	0.60±0.00	-4.89%	-5.00%
	EBT3	0.0882±0.0004	0.0688±0.0003	0.84±0.00	0.63±0.00		
Position 9	EBT	0.0891±0.0001	0.0980±0.0002	0.72±0.00	0.82±0.00	-4.08%	-7.03%
	EBT3	0.0802±0.0001	0.0912±0.0003	0.75±0.00	0.87±0.00		

## Discussion

In the present study, EBT and EBT3 radiochromic films were compared in dosimetry inside typical breast cancer radiotherapy fields. According to the data presented in Table 3 and Table 5 (NOD data from the calibrations and on-RANDO phantom measurements for identical irradiation conditions) NODs that were obtained by EBT radiochromic film are higher than those from the EBT3 radiochromic film. This difference can be attributed to the thickness of the active layers, overall thicknesses of the films and the colors of the films, according to the data in Table 1. In other words, the total thickness of the two active layers in EBT radiochromic film is 34 micrometers and in EBT3 radiochromic film is 30 micrometers. This causes that the NOD of EBT film be more than EBT3 film. From investigation of the scanners light attenuation, the thicker EBT3 radiochromic film leads to higher NOD than the thinner EBT, since more absorption will occur with EBT film. Since the color of each film have effect on its absorption (according to difference in the colors of the films), to show their sensitivity, the films were analyzed in the red color channel. According to the calibration process, the difference between the NODs of two films was measured and reported. The highest difference in NOD is related to the dose of 0.75 Gy which is equal to 14.19%, and the lowest is related to 0.2 Gy which is equal to 3.31%. In a study that was performed by Reinhardt et al[1] on a comparison of EBT2 and EBT3 radiochromic films, NOD values for a specific dose for EBT3 radiochromic film was more than the EBT2 film. This result is consistent with the results of this study, because the sensitive layer and the color of EBT2 and EBT3 radiochromic films are similar, but the overall thickness of EBT2 film is more than EBT3, which causes a higher attenuation by EBT2. Based on a study that was conducted by Brown et al[8] NOD values for a specific dose for EBT radiochromic film was higher than EBT2 and EBT3 radiochromic film. In this case, the thickness of the sensitive layer of EBT film was more than that of the two other films which is also consistent with the results presented in the current study.

In Table 4, doses in various depths are compared for EBT, EBT3 and Semiflex ionization chamber. The lowest difference between the two radiochromic films is occurred in the depth of 0.5 cm from the surface which is equal to 0.07% and its maximum is occurred in the depth of 10 cm which is equals to 5.41%. The responses of two films are almost the same. According to the data in Table 1, this similarity can be closely attributed to the sensitive layers of the films. As it can be seen from Figure 1, the total thickness of the two sensitive layers (active layers) in EBT is 34 microns, where the thickness of the sensitive layer in EBT3 film is 30 microns. In comparison of the films with ionization chamber, the highest percentage difference in surface dose by EBT film is equal to 56.30% and the lowest is occurred in the depth of 1.5 cm which is equal to 2.34%. With EBT3 film, the highest difference with ionization chamber is related to the surface, which is equal to 55.20% and the lowest is for the depth of 2 cm which is equal to 3.2%. The sensitive volume of the ionization chamber is substantially different in comparison to the sensitive layers of the two film types, so large differences are expected between the results of the ionization chamber and films in surface dosimetry. Therefore, ionization chambers are not suitable for surface dosimetry. International Commission on Radiological Protection (ICRP) has suggested the evaluation of skin dose at a depth of 0.07 mm, which is the thickness of basal layer in skin[9]. The inner diameter of Semiflex ionization chamber is 5.5 mm with a sensitive volume of 0.125 cm^3^. This physical structure causes a great difference with the recommended depth for skin dose assessment and therefore it incorporates with large difference compared to the films. Due to the better spatial resolution of TLD over Semiflex chamber, it is suggested that in future studies, TLD be used for surface dosimetry.

As it was shown in Table 5, differences are observed between the obtained doses by EBT and EBT3 radiochromic films in skin dose measurement on RANDO phantom in positions illustrated in Figure 2. In comparison between two radiochromic films for the medial field, the maximum difference is related to position 1 which is equal to 13.51%. The lowest difference is related to the position 4 which is equal to 0.85%. In the lateral field, the maximum difference is related to the position 1 which is equal to 10.62% and the lowest percentage difference is related to the position 4 which is equal to 0.33%. Since the irradiation conditions were the same for these two films, this difference can be attributed to the difference in the fitted functions of the films. Different fitting functions were selected for the two films to have a higher accuracy in the fitting for each film. In other words, the function fitted to each film was chosen based on a closer value of R2 to 1.0. Each of these functions gives the closer value to the actual dose only in a limited dose range, and in the other dose regions there may be errors in the dose results from the fitting function. In different points, the recorded differences by these two films have positive and negative differences. It means that sometimes dose of EBT radiochromic film is higher than EBT3 and sometimes a reverse effect is observed. Additionally, according to Figure 2, in the medial and lateral fields, the greatest and the lowest differences are in the positions 1 and 4, respectively.

In the treatment of breast cancer, the overall dose that is delivered to skin from two treatment fields is important, on the other hand, in this study the dose in each field was examined separately. It is recommended that for more relevant clinical purposes, this study to be performed in such a way that the overall skin dose from two treatment fields to be evaluated during a treatment fraction. In addition, because of the availability of only a male RANDO phantom in our home-institution, in this study a male RANDO phantom was used. However, it should be noted that breast cancer is more common in women and the anatomy of a female phantom is different from that of a male one. This will have impact on the results of skin dose measurements. Therefore, it is suggested that in future studies on the skin dose measurement in breast cancer radiotherapy, a female RANDO phantom be utilized. 

## Conclusion

Based on the obtained results, in the measured dose range (0.2 Gy-4 Gy) in the calibration and on-RANDO phantom measurement steps, NODs of EBT radiochromic film are higher than those of the EBT3 radiochromic film which can be attributed to the different thicknesses of the active layers, the overall thicknesses and the colors of these two film types.

 In the medial and lateral treatment fields of breast cancer radiotherapy, at most of the positions, the doses measured by the EBT3 radiochromic film are higher than those of the EBT film which can be related to the fitted functions used for the EBT and EBT3. The advantage of EBT film over EBT3 is a higher sensitivity, on the other hand, EBT3 film allows the user to use both sides of the film in the scanning process and it is a new version of this film type.

Based on the surface dosimetry results by Semiflex ionization chamber, EBT film and EBT3 film, the sensitive volume of an ionization chamber is substantially larger compared to the films’ sensitive layers, therefore it is not appropriate for surface measurement. 
